# Effects of environmental variables on abundance of ammonia-oxidizing communities in sediments of Luotian River, China

**DOI:** 10.7717/peerj.8256

**Published:** 2020-01-06

**Authors:** Amjed Ginawi, Lixiao Wang, Huading Wang, Bingbing Yu, Yan Yunjun

**Affiliations:** 1 Key Lab of Molecular Biophysics of Ministry of Education, College of Life Science and Technology, Huazhong University of Science and Technology, Wuhan, Hubei, China; 2 Faculty of Marine Science and Fisheries, Red Sea University, Port Sudan, Red Sea State, Sudan

**Keywords:** Luotian river, Ammonia-oxidizing archaea, Functional marker genes, Ammonia-oxidizing bacteria

## Abstract

Ammonia-oxidizing communities play important functional roles in the nitrification. However, environmental stresses can significantly affect this process by controlling the abundant communities of ammonia-oxidizing archaea (AOA) and ammonia-oxidizing bacteria (AOB) communities. In this study, we examined the abundance variations of ammonia-oxidizing communities using quantitative polymerase chain reaction (qPCR) and terminal-restriction fragment length polymorphism (T-RFLP) in a typical subtropical river, Luotian County, South Dabie Mountains, China. Clone libraries were conducted to evaluate the community structure and abundance of AOA and AOB in sediments. Results showed that *Nitrososphaera sp* and *Nitrosopumilus sp* were the most dominant AOA. The abundance of the AOA and AOB amoA gene ranged from 5.28 × 10^8^ gene copies (g-soil^−1^) to 2.23 × 10^8^ gene copies (g-soil^−1^) and 5.45 × 10^8^ gene copies (g-soil^−1^) to 3.30 × 10^7^ gene copies (g-soil^−1^), respectively. Five environmental variables, namely, ORP, DO, NO${}_{3}^{-}$, Temp, and NH${}_{4}^{+}$ were played a major function in microbial communities of AOA and AOB in sediments. The T-RFLP profiles of AOA showed that 488 and 116 bp T-RFs were dominated. Overall, the results of this study showed that anthropogenic activities andenvironmental stress in rivers can alter the structure and function of microbes in their variable environment.

## Introduction

Nitrification modeling in aquatic environments has been improved due to the detection of ammonia-oxidizing archaea (AOA) and ammonia-oxidizing bacteria (AOB), which have been observed as a single group that intercedes in ammonia oxidation (Wang et al., 2017). Microbial nitrification is the permanent oxidation of ammonia (NH_3_) to nitrate (NO${}_{3}^{-}$) through nitrite (NO${}_{2}^{-}$) (Kataoka et al., 2018). The ammonia monooxygenase (*amoA*) gene is a useful indicator of the limiting steps in nitrification and a possible indicator of potential N mineralization (Nelson et al., 2015). As a function target, *amoA* is more useful than 16S rRNA gene in the study of AOA and AOB community structures (Rotthauwe, Witzel & Liesack, 1997). Studies on sediments in malodorous streams conducted by investigating the 16S rRNA clone library and assessing ammonia monooxygenase (*amoA*) genes showed that the number of *amoA* gene copies is higher in AOA than in AOB (Li et al., 2015b; Schleper & Nicol, 2010). Many investigations determined AOA and AOB *amoA* gene sequences and evaluated the proliferation of various ammonia-oxidizing communities in different environments (Bollmann, Bullerjahn & McKay, 2014; Leininger et al., 2006). However, further investigations are needed to explore the effects of environmental parameters on ammonia-oxidizing communities.

Physiochemical factors can influence the abundance and diversity of AOA and AOB microbes in several vital processes, including river N cycling ecosystems (Jiang et al., 2016; Wang et al., 2018). For instance, oxygen availability (Gleeson et al., 2010), phosphorus (Herfort et al., 2007), sulfide (Burgin et al., 2012), pH (Li et al., 2015a), soil type (Huang et al., 2011), NH${}_{4}^{+}$-N, and NO_3_-N (Li et al., 2012) have been classified as significant parameters that affect the distribution and diversity of ammonia-oxidizing communities in different ecosystems (Behrendt et al., 2017). The typical optimum pH for nitrification is 7.8 (Antoniou et al., 1990; Kumar & Nicholas, 1982). However, the pH level is a critical physiochemical factor that affects the distribution of ammonia-oxidizing communities. In natural habitats with high pH values (Sahrawat, 2008; Siliakus, Van der Oost & Kengen, 2017), AOB is less dominant and more sensitive than AOA (Zheng et al., 2017). At present, investigations on AOB and AOA functions in ammonia oxidation have attracted considerable attention from researchers (Cydzik-Kwiatkowska & Zielinska, 2016). The microbial communities of AOA and AOB are active in nitrification and participate in the turnover process of aquatic environments (Falkowski, Fenchel & Delong, 2008). Rivers are characterized by rich geographic and physicochemical ranges that represent a suitable environment for a diverse microbial ecosystem (Fierer & Jackson, 2006). Human activities can significantly affect aquatic ecosystems (Ma et al., 2019), which in turn influence the microbial N cycling processes and lead to environmental deterioration (Li, Zhang & Zhang, 2012). High ammonia concentrations in river streams are toxic to fish and other aquatic organisms (Matson et al., 1997).

Studies on the influence of environmental indicators on the distribution, abundance, and diversity of ammonia-oxidizing communities mainly focused on soil (Zhang et al., 2009), estuaries (Bernhard et al., 2010) and oceans (Zhang et al., 2015). The investigations on rivers (Liu et al., 2013; Zhou et al., 2015) are extremely limited, and data on river sediments are scarce. However, rivers play dominant roles in human life, agriculture, and industry. The ecohealth of river ecosystems is drawing wide attention, especially from the administration, scientists, and environmental protectors.

The Luotian River, which is a typical subtropical river that is also known as the Yishui River, is 87 km long and 80–220 m wide and provides many ecological services for its inhabitants. The Luotian River converges into the Ba River and then into the Yangtze River near Wuhan City, China. The temporal and spatial patterns of the river’s environmental status have considerable differences. The Luotian River basin has a subtropical monsoon climate with rainy summer and winter. The basin has a population of approximately 600,000 people and covers an area of 2,144 km^2^. However, high ammonia nitrogen (NH_3_-N) has been discovered at several locations in the river. The potential influence and mechanisms of ammonia nitrogen metabolism in this river are insufficiently understood. The Luotian River can also be regarded as a model of subtropical rivers and streams in developing countries in Asia because of its characteristics.

Therefore, the aims of the present study are (1) to examine the diversity and abundance of AOA and AOB in the Luotian River by identifying functional marker gene *amoA*, (2) identify environmental factors that influence the abundance of ammonia-oxidizing communities, and (3) determine potential linkage among human activities and the diversity of AOA and AOB communities.

## Materials and Methods

### Physiochemical analysis

The study was conducted at the Luotian River in Huanggang City, South Dabia Mountains, Hubei Province, China (30°35′N, 115°06′E) (Fig. S1). This location has a series of mountains with 70% forest coverage. Five streams originate from the Dabie Mountains and flow in the southwest direction.

The physiochemical parameters of the sediment samples were assessed in triplicate following the methods of a previous work (Bailey et al., 2013). Physiochemical parameters, including oxidation–reduction potential (ORP), total suspended solids (TSS), temperature (Temp), pH, dissolved oxygen (DO), and conductivity (Cond), were determined with a multiparameter probe (YSI Professional Plus). Moreover, magnesium (Mg), calcium (Ca), nitrate (NO${}_{3}^{-}$), ammonia (NH${}_{4}^{+}$), nitrite (NO${}_{2}^{-}$), total nitrogen (TN), sulfate (SO${}_{4}^{-2}$), free cyanide (CN), and phosphorus (PO${}_{4}^{-3}$-P) were also analyzed with an electronic spectrophotometer (DR2800, Hach Company, Loveland, CO, USA).

### Samples collection

Three replicate samples were collected from each sampling site at the Luotian River in May, August, and October 2018. Nine stations (S1–S9), specifically, pristine upstream (S1–S4), anthropogenic activities in Luotian City (S5–S7), downstream located far from human activities (S8), and industrial zone for waste discharge (S9), were selected for this study (Fig. S1). A total of 50–100 g sediments were collected in triplicate from each site at a 0–5 cm depth, transferred to sterile impermeable plastics with liquid nitrogen and then transported to the laboratory. All samples were conserved at −80 °C before DNA extraction.

### Nucleic acid extraction, PCR, cloning, and sequencing

Microorganisms from the sediments were studied by extracting DNA using a FastDNA Spin Kit for Soil (MP Biomedicals, Solon, OH, USA). A NanoDrop (2000c) spectrophotometer (Wilmington, DE, USA) was used to quantify DNA according to the manufacturer’s instructions. Thermal cycling conditions for each primer set and primers’ names are shown in Table S1.

PCR amplification was performed with a peqSTAR 2 × double block thermocycler (PEQLAB Biotechnologies, Erlangen, Germany) with the following conditions: 50 µL mixture comprising 25 µL 2 × Master Mix (Thermo Fisher Scientific, USA), 0.2 µM of each primer, and 50–100 ng DNA. Thermal cycling was performed in an initial denaturation step for 5 min at 95 °C. The sizes of the PCR amplification products were analyzed by 1% gel electrophoresis.

The clone libraries of AOA and AOB *amoA* gene-amplified fragments from each sampling site were obtained on the basis of a previously reported study (Jin et al., 2011). After the extraction of DNA and PCR products of the *amoA* genes, the mixture of PCR products was briefly gel-purified and cloned with a pMD19-T cloning kit (TaKaRa Biotechnology, Wuhan, China). A total of >30 clone libraries were randomly tested for the positive insertion of DNA products by PCR amplification with primer M13F and M13R. Then, these libraries were sequenced using an ABI 3,730 × 1 sequencer (Wuhan, China). The DNA sequences were analyzed and edited with BioEdit software version 5.07 and MEGA version 7.

### Phylogenetic analysis

The sequences were first cropped. Then, the alignments were completed by ClustalW and analyzed by BLASTn according to the nonrepetitive National Center for Biotechnology Information (NCBI) database (Morgulis et al., 2008). Operational taxonomic units (OTUs) were determined as clusters, where the sequence identities were performed (>90%) by the Ribosomal Database Project (RDP) (http://rdp.cme.msu.edu and http://www.mothur.org). Phylogenetic trees were created using MEGA 7, the neighbor-joining method of reference (Saitou & Nei, 1987) (http://www.phylogeny.fr), and FigTree v1.4.3.

**Figure 1:**
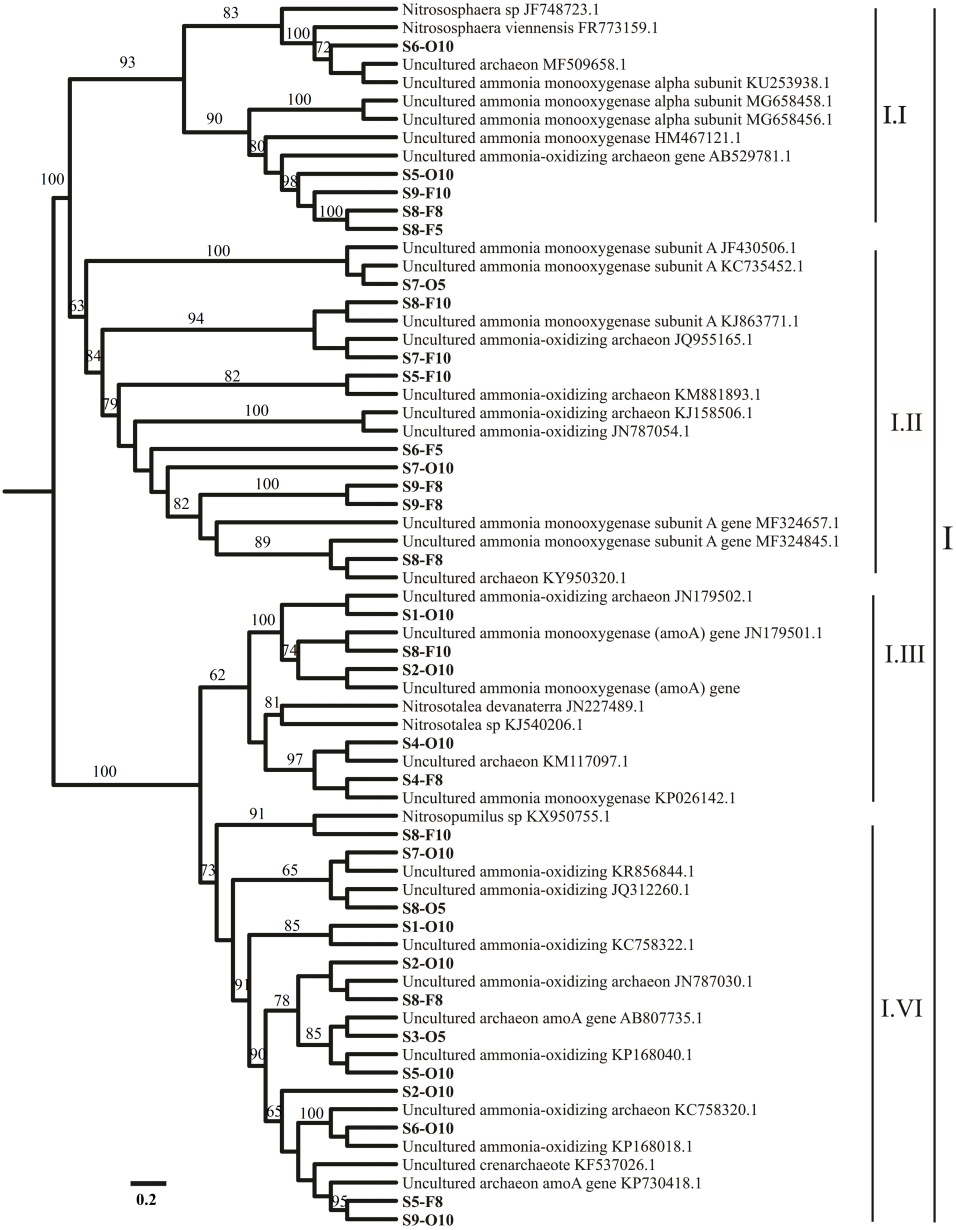
Representation of phylogenetic relationships of AOA gene sequences through neighbor joining. Bootstrap supported values were 1,000 replicates (values larger than 60% were indicated). The scale bar represents 0.2; evolutionary analyses were implemented on MEGA 7.

AOA and AOB *amoA* communities were partially sequenced from the nine stations (S1–S9). Among 109 archaeal *amoA* clone libraries, 54 archaeal *amoA* genes were analyzed. As a result, 34 operational taxonomic units (OTUs) were recovered on the basis of a 1 amino acid residue cut-off (Fig. 1, Table S3). Among 83 bacterial *amoA* clone libraries, 61 bacterial *amoA* sequences were examined. Only 3 OTUs were registered on the basis of a 1 amino acid residue cut-off (Fig. S2, Table S3). BLASTn analysis identified that most sequences were close to the *amoA* genes of uncultured bacteria. Ammonia monooxygenase genes were inserted into the dataset for the most similar GenBank sequences according to the BLASTn results. Sequence alignment was performed. The phylogenetic trees of ammonia-oxidizing archaea and bacteria are presented in Fig. 1 and Fig. S2, respectively. A neighbor-joining tree was designed using the *amoA* gene sequences and the sequences related to those accessed from GenBank. All the OTU sequences of AOA and AOB were deposited in the NCBI database under accession numbers MG238515-MG238543 and MG519671-MG519707.

### Quantitative PCR

Real-time PCR was conducted to measure the abundance of functional marker genes in the sediments. 16S rRNA gene copies were measured using SYBR Green I Real-Time PCR. Gene abundance was measured in triplicate by amplifying the 16S rRNA gene using specific primers and PCR conditions, as shown in Table S1 (Keshri, Mishra & Jha, 2013). Each of AOA and AOB samples was quantified by an ABI Prism sequence detection system on the basis of the SYBR Green I method. The primer sets and thermal cycling conditions for the qPCR are described in Table S1. Each reaction was performed in 25 µL of reaction mixtures containing 12.5 µL of SYBR Premix Ex Taq (TaKaRa Biotechnology, Wuhan, China), 1 µL each of forward and reverse primers, and 1 µL DNA template. The specified PCR for each target gene was verified by the generated melting curves and agarose gel electrophoresis. All assays were conducted in triplicate with 85%–95% efficiency. Cycle thresholds were made in comparison with standard curves that were constructed using 10-fold 1:10 serial dilution from 10^1^ to 10^8^ gene copies per gram of dry weight. The *R*^2^ values for the normal curves were ≥0.993 for all tests.

### AOA and AOB Investigation by terminal-restriction fragment length polymorphism (T-RFLP)

T-RFLP was used to investigate the PCR products of AOA and AOB (several tests were removed from the AOA and AOB investigations because of inadequate PCR output). Fluorescence-labeled PCR products were obtained by applying forward primer 5′-labeled with 6-carboxyfluorescein for the AOA and AOB genes (Table S1) under the following cycling conditions: initial denaturation at 95 °C for 5 min, followed by 30 cycles of denaturation at 95 °C for 30 s, annealing at 54 °C for 30 s, and extension at 70 °C for 60 s.

Each 50 µL of the PCR reaction contained 10 X ThermoPol reaction buffer, 1.5 mM MgCl_2_, 0.1 mM dNTPs, 0.25 µL of Taq DNA polymerase (New England Biolabs Ltd., China), 0.2 µg µL^−1^ of bovine serum albumin (Roche, Penzberg, Germany), 0.1 µM of each primer (Table S1), and 1 µL of the DNA template. The PCR fragments were analyzed by gel electrophoresis to confirm the amplification of a specific area. After purification, amplicons were selected to digest the fluorescence-labeled PCR products into two separate enzymatic digestions using *HhaI* and *MspI* for bacteria and *HpyCH4V* and *RsaI* for AOA. The amplicons were processed for 2 h in a thermocycler at 37 °C and 65 °C.

Restriction products were purified using ethanol precipitation (Sambrook & Russell, 2006) and resuspended in 20 µL of water. Then, 10 µL of aliquots was mixed with 13 µL of HiDi formamide (Applied Biosystems) and 0.3 µL of internal DNA fragment size standard GeneScan-500 LIZ (Applied Biosystems). The product was analyzed with an ABI PRISM 3730 Genetic Analyzer (Applied Biosystems). T-RFs were defined by GeneMarker Demo software (V 1.91, Applied Biosystems). Products within 40 and 600 bp were combined in the investigation. The peak area was represented as a percentage of the entire peak area, the profile of <1.5% of the entire area was excluded from the study.

### Statistical analysis

Principal component analysis (PCA) was conducted to describe whether environmental variables can be performed to discriminate among sampling sites in the Luotian River. PCA was carried out using PAST v.3.15 (Hammer, Harper & Ryan, 2001). To determine the environmental indicators that best explained the variation in ammonia-oxidizing communities, redundancy analysis (RDA) was carried out using Canoco version 4.5 for Windows (Microcomputer Power, Ithaca, New York, United States) (Ramette, 2007). Spearman correlation matrix was used to describe the relationships among environmental indicators and AOA and AOB (Cassman et al., 2019). A *p* <0.01 and *p* <0.05 were considered significant, and the matrix was created using Excel add-in XLSTAT 2017 (Fig. S3). The algorithm in BLASTn was used to define the most similar sequences. The aligned sequences were converted into amino acids by applying MEGA 7 and used for the assembly of phylogenetic tree neighbor-joining by using Poisson model distances and pairwise deletion of gaps and missing data. Bootstrapping (1,000 replicates restructured) was performed for phylogenetic restructuring.

## Results

### Physical and chemical characteristics of Luotian River

The environmental parameters indicated that the sampling sites were influential, as shown by the variability of many ecological features (Table 1). The Cond, SO${}_{4}^{-2}$, Temp, NH${}_{4}^{+}$, NO${}_{3}^{-}$, NO${}_{2}^{-}$, and TN in S9 were relatively higher than those recorded in other stations. Low pH and ORP was also observed in S4 and S9, respectively. The other environmental parameters, although varied, did appear consistent between study periods or sampling sites.

**Table 1:** Physical and chemical parameters of Luotian River (mean of triplicate samples standard deviations).

	**Unit**	**S1**	**S2**	**S3**	**S4**	**S5**	**S6**	**S7**	**S8**	**S9**
DO	mg L^−1^	9.26 ± 0.01	9.24 ± 0.01	9.53 ± 0.02	11.62 ± 0.01	8.33 ± 0.1	6.45 ± 0.02	11.31 ± 0.01	7.73 ± 0.01	5.7 ± 0.02
pH	–	8.52 ± 0.24	7.1 ± 0.21	7.8 ± 0.1	6.58 ± 0.1	7.6 ± 0.001	7.3 ± 0.001	8.73 ± 0.1	7.73 ± 0.001	7.49 ± 0.001
NH${}_{4}^{+}$	mg L^−1^	0.043 ± 0.127	0.013 ± 0.01	0.083 ± 0.01	0.087 ± 0.01	0.02 ± 0.001	0.06 ± 0.001	0.15 ± 0.01	0.06 ± 0.001	0.68 ± 0.21
CN	mg L^−1^	0.001	0.001	0.001	0.001	0.001	0.001	0.005	0.001	0.003
SO${}_{4}^{-2}$	mg L^−1^	9.33 ± 0.52	9 ± 0.43	9.33 ± 0.2	15 ± 0.1	7.66 ± 0.01	9.33 ± 1.02	13 ± 0.1	15.3 ± 0.6	16.67 ± 1.95
NO${}_{2}^{-}$	mg L^−1^	0.009	0.031	0.009	0.006	0.005	0.009	0.02	0.033	0.375
NO${}_{3}^{-}$	mg L^−1^	0.45 ± 0.124	0.31 ± 0.177	0.39 ± 0.02	0.023 ± 0.013	0.12 ± 0.125	0.033 ± 0.02	0.35 ± 0.01	0.36 ± 0.01	0.47 ± 0.01
TN	mg L^−1^	1.97 ± 0.468	1.6 ± 0.177	0.65 ± 0.03	1.9 ± 1.34	0.87 ± 0.2	3.63 ± 1.03	1.67 ± 0.234	1 ± 0.815	7.97 ± 1.21
Mg	mg L^−1^	0.74 ± 0.08	1.38 ± 0.03	1.2 ± 0.01	0.76 ± 0.174	1.4 ± 0.03	1.36 ± 0.124	1.37 ± 0.001	1.28 ± 0.01	2.79 ± 1.12
Ca	mg L^−1^	1.5 ± 0.031	2.4 ± 0.231	2.395 ± 0.12	2.596 ± 0.11	2.73 ± 0.02	2.75 ± 0.14	2.67 ± 0.01	3.02 ± 0.01	1.19 ± 0.21
TSS	mg L^−1^	1 ± 0.02	2 ± 0.1	1 ± 3.412	1 ± 1.541	4.33 ± 1.31	0.67 ± 0.42	12.33 ± 2.01	5 ± 2.31	11 ± 3.66
ORP	mV	141.63 ± 3.89	137.33 ± 3.22	132.03 ± 4.26	124 ± 4.51	91.43 ± 11.18	103.57 ± 5.57	67.03 ± 1.43	92.83 ± 1.47	−65.067 ± 1.5
Cond	µS cm^−1^	65.07 ± 5.79	130.67 ± 5.97	131.67 ± 5.65	96.37 ± 4.61	119.5 ± 3.14	137.17 ± 3.84	158.53 ± 3.2	163.83 ± 4.01	612.67 ± 14.11
P	mg L^−1^	7.47 ± 0.003	7.537 ± 0.001	7.539 ± 0.001	7.54 ± 0.003	7.605 ± 0.001	7.599 ± 0.001	7.606 ± 0.001	7.617 ± 0.001	7.59 ± 0.001
Temp	°C	17.4 ± 0.33	19 ± 0.1	20 ± 0.01	20.73 ± 0.77	19.75 ± 0.02	19.8 ± 0.01	21.7 ± 0.01	19.7 ± 0.32	21.96 ± 0.41

**Notes.**

DO dissolved oxygen CN free cyanide TSS total suspended solids ORP oxidation–reduction potential Cond conductivity TN total nitrogen P phosphorus Temp temperature mV milli-volts

### Archaeal and bacterial amoA-based community structures

Most of the sequences were related to *Nitrososphaera* and *Nitrosopumilus* (three AOA clusters, I.I, I.II, and I.IV). However, the sequences connected to *Nitrosotalea* cluster I.III were noted in S4 (Fig. 1). Only 1 AOB cluster was related to genus *Nitrosomonas* (Fig. S2).

### Relationships among physicochemical variables and sampling sites

**Figure 2:**
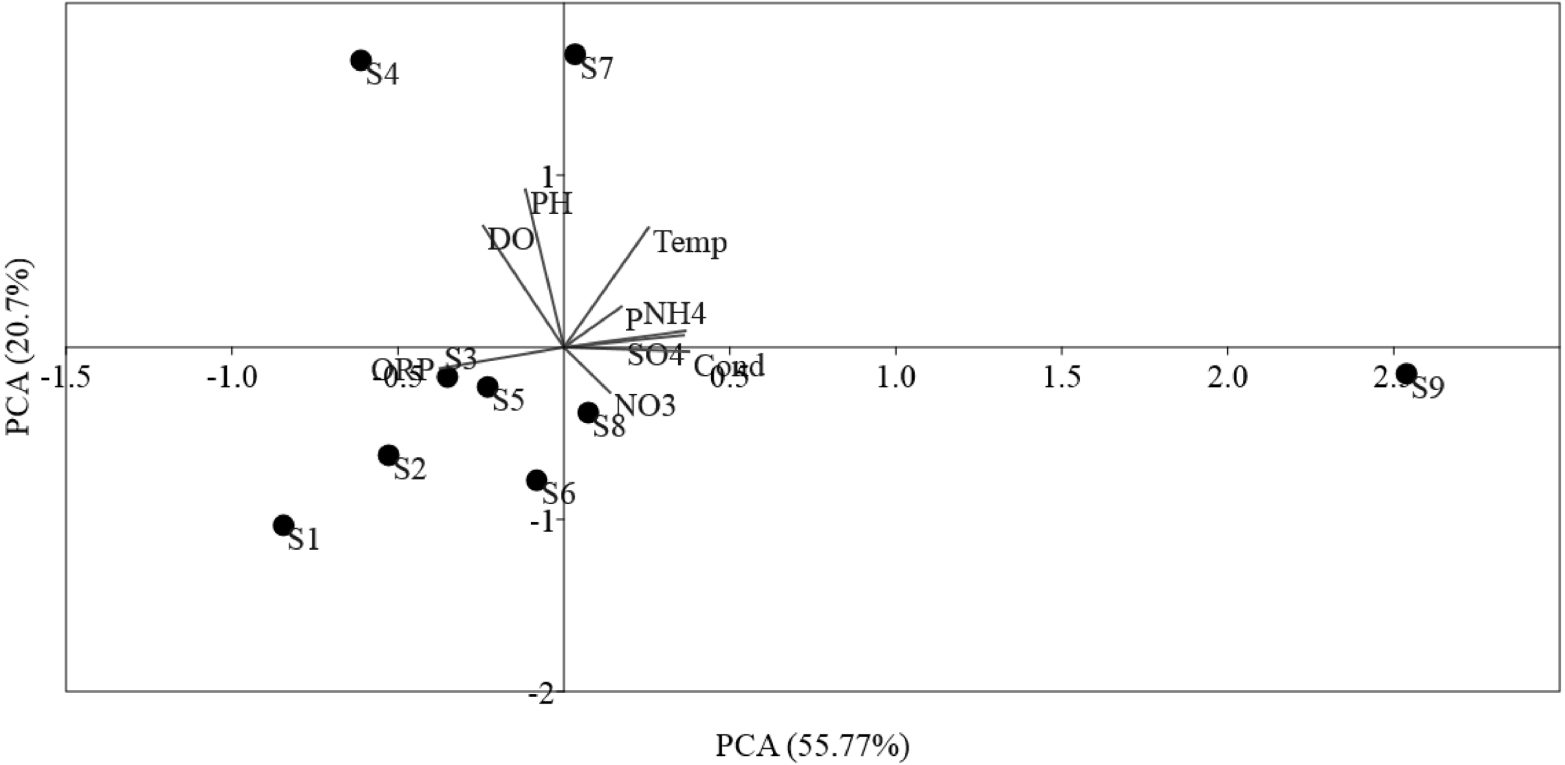
Ordination plots showing PCA of environmental variables in Luotian River, China. The environmental variable axes are represented by the arrow lengths (circles indicate sampling sites and arrows show environmental variables).

The ordinations of physicochemical variables obtained by PCA were differentiated between sites (Fig. 2). The sampling sites were disseminated along PCA 1, which described 55.77% of the environmental data. PCA 1 was positively correlated (*p* < 0.01) with NO${}_{3}^{-}$, Temp, NH${}_{4}^{+}$, SO${}_{4}^{-2}$, and phosphorus, in addition to, negatively correlated (*p* < 0.05) to DO, ORP, and pH, which mostly indicated anthropogenic activities in the sampling sites. PCA 2 described 20.7% of the environmental data and was positively correlated (*p* < 0.01) to Temp and negatively correlated (*p* < 0.05) to ORP, pH, and DO. PCA showed the locations S9 within the Luotian River, that is, most of the sampling sites were clarified by the presence of anthropized activities due to the industrial zone for waste discharge in the river.

**Figure 3:**
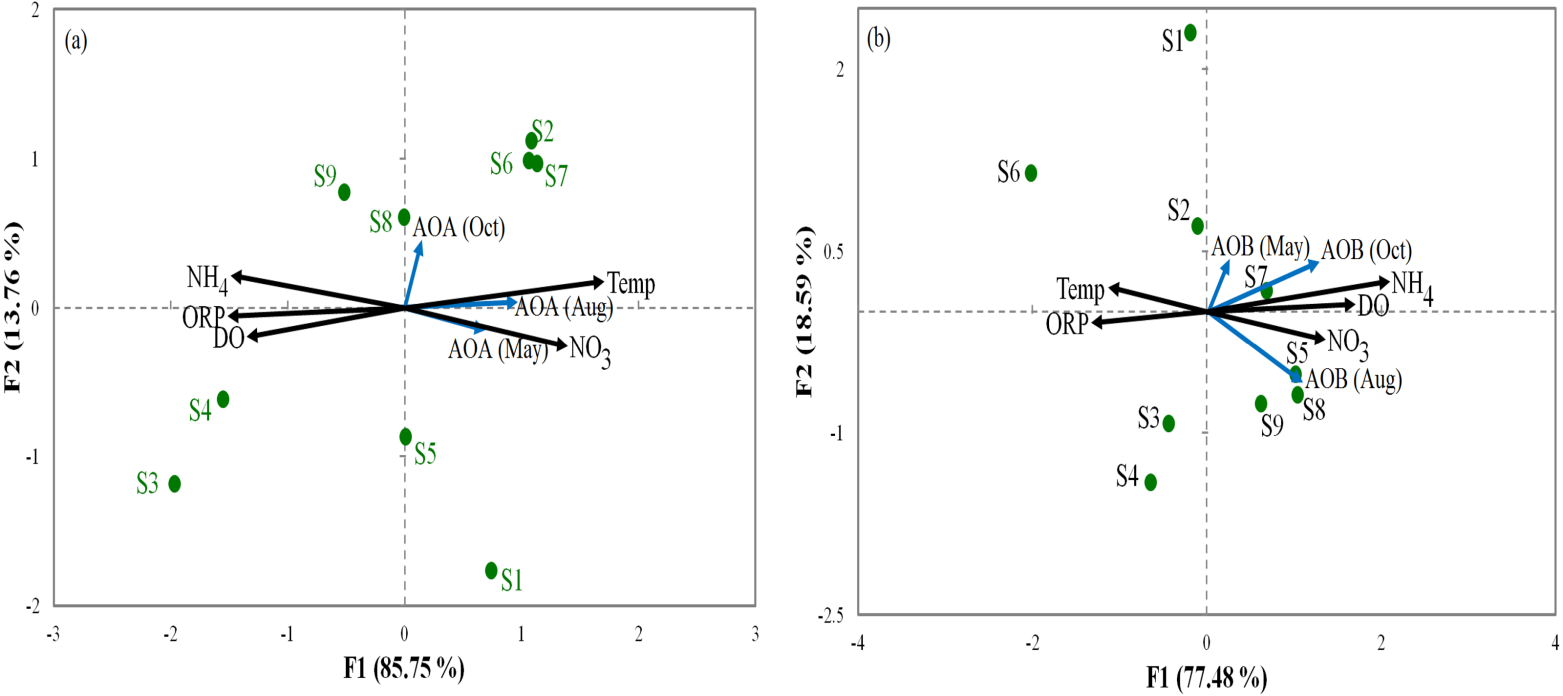
Redundancy analysis ordination plots of correlations between the communities of AOA (A) and AOB (B) and environmental factors from the sediment samples at the nine sites. Correlations between redundancy analysis (RDA) and environmental variables axes are represented by the lengths of arrows (circles: sampling sits arrows, environmental variables). Only analyses that showed significant Spearman corrected *p*-values are included.

Relationships between the abundance of ammonia oxidization genes (AOA and AOB) and the environmental parameters were analyzed by RDA (Fig. 3). For AOA, axis1 and axis2 explained 85.75% and 13.7% of the total variation, respectively, in community structure (Fig. 3A). For AOB, axis1 and axis2 respectively explained 77.48% and 18.59% of the total variation (Fig. 3B). Our results show that ORP had important influences on AOA and AOB communities in all sediments. Furthermore, the overall composition of the AOA and AOB communities was negatively correlated (*p* < 0.05) with ORP (Fig. 3). However, NO${}_{3}^{-}$ was positively correlated (*p* < 0.01) with the overall composition of the AOA and AOB. The order of the dominant factors corresponding to the levels of impact on the ammonia monooxygenase communities was ORP, Temp, NO${}_{3}^{-}$, DO, and NH${}_{4}^{+}$.

Spearman correlation matrix was calculated to investigate the correlations among environmental factors (Table 1) and abundance of AOA and AOB. All correlation analyses were applied to evaluate the significant number of relations at *p* <0.01 and *p* <0.05 (*p*-values). The results shown in Fig. S3 indicated that AOA and AOB communities were positively correlated (*p*<0.05) with DO, ORP, and NO${}_{3}^{-}$ and negatively correlated (*p* < 0.01) with Temp and NH${}_{4}^{+}$.

### Abundance of functional ammonia-oxidizing communities in sediments

**Figure 4:**
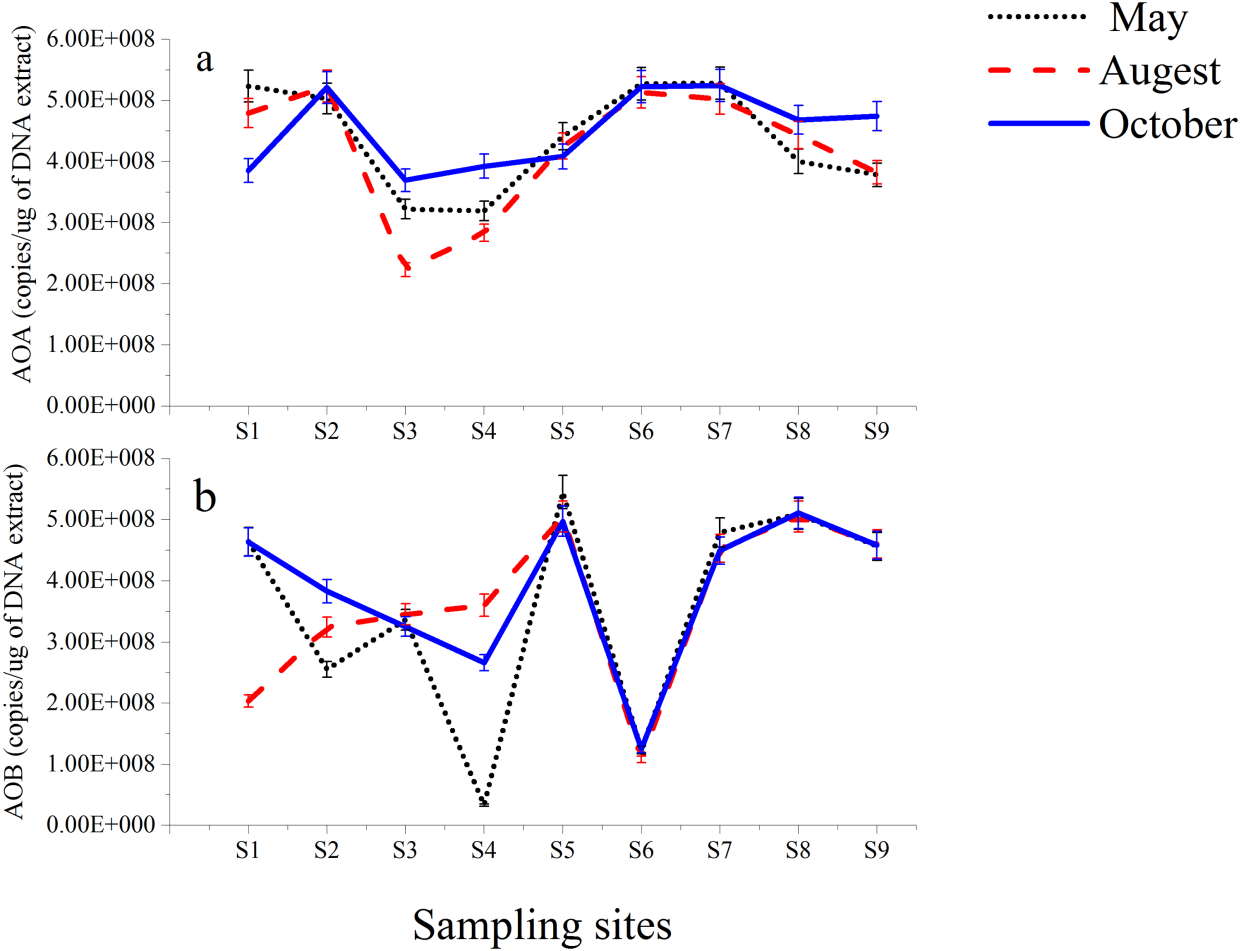
Abundance of AOA (A) and AOB (B) gene copies in sediments from Luotian River sampling sites. Standard errors were estimated from the analysis of triplicate qPCR samples.

The functional genes (*amoA* genes) of AOA and AOB from nine different stations along the Luotian River were investigated. The results showed insignificant variations in the copy number of the total microorganisms, as evaluated on the basis of 16S rRNA (Table S2). AOA *amoA* copy numbers ranged from 5.28 × 10^8^ gene copies (g-soil^−1^) from S7 (May) to 2.23 × 10^8^ gene copies (g-soil^−1^) from S3 (August). AOB *amoA* copy numbers ranged from 5.45 × 10^8^ gene copies (g-soil^−1^) from S5 (May) to 3.30 × 10^7^ gene copies (g-soil^−1^) from S4 (May). However, high numbers of gene copies in AOA and AOB (Fig. 4) were observed at S7 and S5; they were calculated to be 5.28 × 10^8^ and 5.45 × 10^8^ gene copies (g-soil^−1^) from May, respectively. In most of the sampling sites, higher numbers of gene copies were presented by the 16S rRNA group than by the AOA group (Table S2). S2, S6, and S7 showed higher numbers in AOA than in AOB, whereas at S5 and S8, AOB was higher than AOA. However, the abundance of AOB at S4 from May showed lower numbers of gene copies of 3.3 × 10^7^ than the other sites (Fig. 4B).

### T-RFLP profiles of ammonia-oxidizing communities

**Figure 5:**
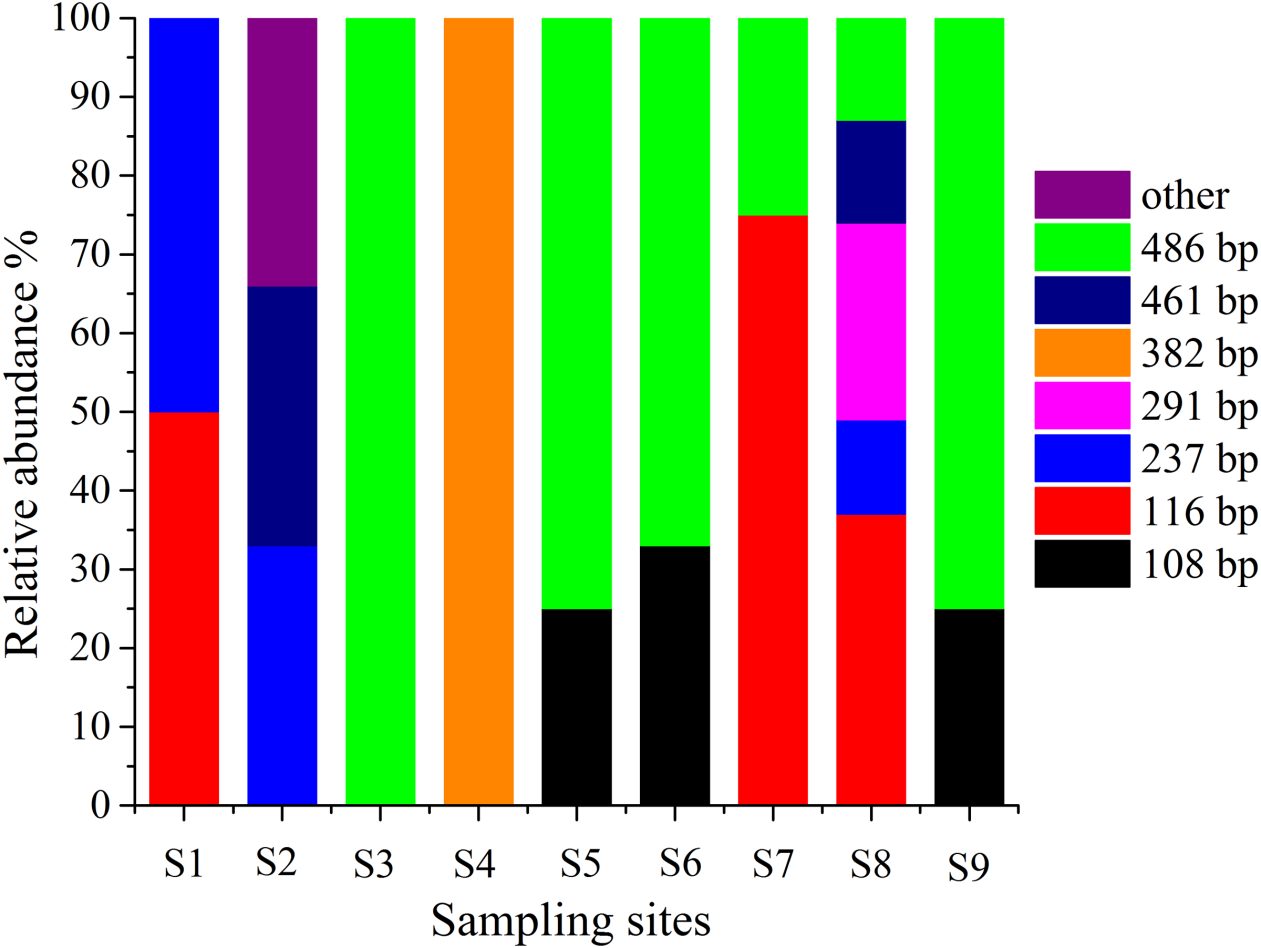
Terminal-restriction fragment length polymorphism (T-RFLP) of AOA during study periods across different treatments and particle-size fractions. The relative abundance of each AOA was described through the corresponding standardized T-RFLP peak.

T-RFLP is an essential method for detecting microbes in the environment, and it can be used to observe the abundance and composition of microbial communities in a statistical manner (Lueders & Friedrich, 2003). Therefore, T-RFLP is an effective tool to determine the diversity and abundance of the AOA group in and around the sampling locations in this work. The T-RFLP investigation of the AOA group by *RsaI* and *HpyCH4V* enzymes resulted in 8 distinct terminal restriction fragments (TRFs) across all the treatments and sediment fractions. In each sampling site, TRF-116 and TRF-486 were identified in two dominant genotypes (Fig. 5). All 8 T-RFs may be related to the clones of the AOA gene clone library, as shown in Table S4. The T-RFs of 116 and 486 bp were correlated with AOA in *Nitrososphaera* and *Nitrosopumilus*. However, the T-RF of 382 bp was mostly obtained from AOA in *Nitrosotalea*. At S3 and S5–S9, the relative abundances of *Nitrososphaera* and *Nitrosopumilus* were shown by the T-RF of 486 bp. The abundance of AOA in *Nitrosotalea* sp. was shown by the T-RF of 382 bp. Similar T-RFs in various tests appeared from different AOA groups while the T-RFs of 116 and 237 bp were collected by AOA in three groups with various ratios. The diversity of AOA groups between the sampling sites was intricate.

## Discussion

The present study focused on the abundance of ammonia-oxidizing communities and the effects of human activities and environmental parameters on AOA and AOB communities along the Luotian River in China (from upstream to downstream). To the best of our knowledge, this study is the first to investigate the effects of anthropogenic activities and environmental parameters on ammonia-oxidizing communities in this area. Nine sampling sites were selected to investigate the effects of environmental parameters on ammonia-oxidizing communities (Table 1). The physicochemical parameters of the rivers varied among the evolutionary stages. For instance, the physicochemical properties of S1, S2, and S3 sites (pristine upstream) were more suitable than those of S9 (discharge of wastewater treatment plants), whereas the general environmental qualities of S4, S6, and S8 (anthropogenic activities in Luotian City) showed an irregular pattern. Therefore, further investigations are needed to prove these relationships.

The phylogenetic analysis of the AOA *amoA* communities in the sediments showed that most of the revealed sequences (90%–97%) in all sampling sites (S1–S9) were linked to *Nitrososphaera viennensis* and *Nitrosopumilus sp*. clusters I.I, I.II, and I.IV (Lu et al., 2019). *N. viennensis* and *Nitrosopumilus sp*. (moderate) clusters I.I, I.II, and I.IV (Fig. 1) were extracted from the sediments and can autotrophically or mixotrophically be involved in ammonia oxidation in freshwater and sediment environments (Liu et al., 2015a). Yang et al. (2013) reported that *Nitrososphaera*-like microbes are the major AOA groups in freshwater sediments in the Qinghai–Tibetan Plateau. The present results agreed with a previous investigation that focused on *amoA* gene abundance and diversity (Kataoka et al., 2018). In the present study, AOA was related to *Nitrososphaera* subclusters that might have played a significant role in the ammonia oxidation of the sediments from the sampling sites; most OTUs were grouped within one cluster in *Nitrososphaera*. The results showed that most of AOA *amoA* sequences were dominated by clusters (I.I, I.II, and I.IV) in neutral pH (Fig. 1). Therefore, the results agreed with the finding of a previous report (Taylor et al., 2017). *N. viennensis* is enriched by the availability of ammonia (Tourna et al., 2011), and *N. gargensis* is commonly observed in subtropical zones (Kim et al., 2012). The environmental acidic pH has a variety of AOA that is correlated with *Nitrosotalea* (Gubry-Rangin, Nicol & Prosser, 2010). In the present study, the sequences connected to *Nitrosotalea* cluster I.III were revealed at S4, where the pH was 6.5 (Table 1). The results confirmed that *Nitrosotalea devanaterra* cannot be grown under neutral pH (Lehtovirta-Morley et al., 2016) and that acidophilic ammonia oxidation hints the physiological mechanisms in *N. devanaterra*, which had the greatest substrate affinity for AOA (Lehtovirta-Morley et al., 2011). This phenomenon contradicted the results of a previous study (Liu et al., 2013), which showed that the acidophilic AOA *Nitrosotalea devanaterra* and *Nitrosotalea sp*. live in an optimal pH ranging from 4 to 5 (Gubry-Rangin et al., 2011) or at a pH of 6.5, as confirmed in the present study. Nevertheless, the findings of the present study suggest that several species that may be adapted to AOA, that is, *Nitrosotalea* cluster I.III (Fig. 1), can resist a comparably acidic pH (>6.5). Regarding the physiological adjustment of microbial guilds to environmental stress (Li et al., 2018), most of the OTUs found in the sampling sites (more neutral pH) were correlated with I.I, I.II, and I.IV clusters (Ramanathan et al., 2017). Meanwhile, AOB is generally found in river ecosystems (Zhao et al., 2014); the results showed that only one cluster can be related to *Nitrosomonas,* which are identified in aquatic habitats (Hu et al., 2010). *Nitrosomonas* is generally found in environments with high NH_4_ levels, which often indicate anthropogenic activities in the sampling positions, such as wastewater treatment plants (Merbt et al., 2015). A total of 61 sequences were collected and clustered into 3 OTUs among all the AOB genes. As previously reported, this result is due to a high similarity (99%) (Fujitani et al., 2015). In many strains, the clone libraries and gel electrophoresis analysis showed the presence of unspecific PCR fragments, which may be due to the presence of many binding efficiencies of two forward primers to DNA templates (Table S1) (Jin et al., 2011).

PCA was conducted to evaluate the effect of environmental parameters in the sediment samples. PCA1 and PCA2 accounted for 55.77% and 20.7% of the variations, respectively (Fig. 2). The primary factors NO_3_-N, SO${}_{4}^{-2}$, P, Temp, and NH_4_-N had various influences on the sediment samples, which mostly indicated the anthropogenic activities of the sampling sites, as confirmed in previous studies (Schullehner, Stayner & Hansen, 2017; Tong & Xu, 2012). However, the negative correlation of DO and pH can be explained by the fact that the water of the Luotian River has high organic concentration matter that consumes the DO S9 that are in contact with pollution due to wastewater discharge in the river basin. Wastewater commonly contains carbohydrates, proteins, and lipids that decrease DO levels due to anaerobic fermentation and the production of organic acids and ammonia due to the decrease in pH in the river basin (Dutta, Dwivedi & Suresh Kumar, 2018). These influences can be elucidated by the discharge of waste treatment plants (S9). However, PCA showed that DO, ORP, and pH were factors that negatively influenced the environmental parameters in the pristine upstream (Gao et al., 2003; Gleeson et al., 2010). These data further confirmed an inverse relationship between ORP and P (Li et al., 2016). Meanwhile, RDA results showed that AOA and AOB communities were positively correlated with NO${}_{3}^{-}$ and negatively correlated with ORP (Fig. 3). Here, our results suggest that NO${}_{3}^{-}$ andORP were played a vital function in controlling the community composition of AOA and AOB (Li et al., 2012; Liu et al., 2015b). RDA analysis showed that dissolved oxygen is powerful parameters influencing the dynamics of AOB functional genes (Fig. 3B). This agrees with a previous study (Wang et al., 2012), which suggested that DO is one of the most correlated factors in the AOB gene community. Out of all these indicators, ORP, DO, NO${}_{3}^{-}$, Temp, and NH${}_{4}^{+}$, which perform a major function in microbial nitrification in sediments, were confirmed as factors affecting the major environmental and changeable characteristics in this study (Gleeson et al., 2010; Li et al., 2012; Ma et al., 2019). Overall, PCA and RDA suggested that the quality of water and communities of AOA and AOB may change under anthropogenic activities. Wastewater discharge was not addressed in this study.

AOA and AOB communities appeared in many locations and periods. The gene copy ratios of AOA were higher than those of AOB in S2, S6, and S7 (Fig. 4), as confirmed by previous investigations (Tzanakakis et al., 2018). In S5 and S8, the AOB genes were slightly more numerous than the AOA genes, as reported by previous studies on lakes (Bollmann, Bullerjahn & McKay, 2014), estuaries (Li et al., 2015b), and sediments (Huang et al., 2019). The results showed that the higher numbers of gene copies in AOA and AOB were observed from May in S7 and S5, which were 5.28 × 10^8^ and 5.45 × 10^8^ gene copies (g-soil^−1^), respectively. Despite the pH level (6.5) in S4, the abundance of AOB was generally lower (3.30 × 10^7^ gene copies from May) than that in the eight other sampling sites (Fig. 4B). Considering the consequential decrease of pH in the bioavailability of N, acidic environments can encourage AOA growth over AOB because of their significant correlation with ammonia (Li et al., 2015a; Ramanathan et al., 2017). Various results of the comparative abundance of AOB and AOA have been reported in the context of sediments (Kataoka et al., 2018; Yan et al., 2018).

The T-RFLP results showed eight peaks of the AOA population, which significantly changed in various stages. The T-RFLP profiles of AOA represented two dominant T-RFs of 488 and 116 bp (Fig. 5) (Zhang et al., 2011). This result suggested that the AOA T-RFs of 488 bp were the common AOA in all stations. The abundance of peaks is an indication of species and their diversity (Coolen et al., 2007). The total T-RFs were significantly less than the OTUs in the AOA clone library. This condition indicated that the technique was inadequate because the AOB sequences collected in this investigation were digested with restriction enzymes. However, many sequences cannot be separated correctly through enzymes (Jin et al., 2011). Thus, T-RFLP is not an effective approach to examine the diversity of the AOB group in this investigation.

## Conclusion

Ammonia-oxidizing communities and environmental variables differed remarkably among the sampling sites of the Luotian River. In most stations, the AOA genes were more abundant than the AOB genes, thereby reflecting the impact of environmental parameters on the abundance of both microorganisms. The abundance of ammonia monooxygenase (*amoA*) genes was higher in AOA at S2, S6, and S7 compared with AOB. The results also indicated that several species classified into *Nitrosotalea* cluster might be adapted to low acidic pH value (<6.5). Five environmental variables; ORP, Temp, NO${}_{3}^{-}$, DO, and NH${}_{4}^{+}$, have been confirmed as primary environmental indicators on ammonia-oxidizing communities in this study. An inverse relationship between ORP and P was confirmed. Our findings showed the significance of environmental variables in human activities. The discharge of waste treatment plant (S9) underwent greater oscillation than in the pristine upstream, which may be due to the effect of the wastewater. Policies such as decreasing wastewater discharge must be seriously considered to protect the environmental health of the river.

##  Supplemental Information

10.7717/peerj.8256/supp-1 Supplemental Information 1 Supplemental information Figure S1. Representation of phylogenetic relationships of ammonia-oxidizing bacteria (AOB) gene sequences through neighbor joining. Bootstrap supported values were 1000 replicates (values larger than 60% were indicated). The scale bar represents 0.2; evolutionary analyses were implemented on MEGA 7, Figure S2. Representation of Spearman correlation matrix was calculated between the gene copy numbers of 16S rRNA, AOA, and AOB and physicochemical parameters. The colors of the scale bar indicate the nature of the correlation with 1 denoting perfect positive correlation (green), and -1 denoting perfect negative correlation (red) were tested at *p* < 0.01 and *p* < 0.05. The used physicochemical data of the samples applied to Xlstat (www.xlstat.com), Table S1. The PCR primer sets and thermal profiles, Table S2. The gene copy numbers of 16S rRNA, AOA, and AOB in sampling sites. Values and standard deviations were estimated from triplicate an analysis within a single qPCR, Table S3. T-RF fragments and their corresponding clones of AOA.

10.7717/peerj.8256/supp-2 Figure S1 Figure S1 A schematic showing the locations of the sampling sites in the Luotian River; pristine upstream (S1–S4); human influences in Luotian city (S5–S7); downstream, located far from anthropogenic activities (S8); and discharge of waste treatment plant (S9), Map data ©2019 Google.

10.7717/peerj.8256/supp-3 Figure S2 Representation of phylogenetic relationships of ammonia-oxidizing bacteria (AOB) gene sequences through neighbor joining Representation of phylogenetic relationships of ammonia-oxidizing bacteria (AOB) gene sequences through neighbor joining. Bootstrap supported values were 1000 replicates (values larger than 60% were indicated). The scale bar represents 0.2; evolutionary analyses were implemented on MEGA 7.

10.7717/peerj.8256/supp-4 Figure S3 Representation of Spearman correlation matrix was calculated between the gene copy numbers of 16S rRNA, AOA, and AOB and physicochemical parameters Representation of Spearman correlation matrix was calculated between the gene copy numbers of 16S rRNA, AOA, and AOB and physicochemical parameters. The colors of the scale bar indicate the nature of the correlation with 1 denoting perfect positive correlation (green), and -1 denoting perfect negative correlation (red) were tested at p¡0.01 and p¡0.05. The used physicochemical data of the samples applied to Xlstat (www.xlstat.com).

## Additional Information and Declarations

### Data Availability

The following information was supplied regarding data availability: The raw data is available at GenBank: MG238515–MG238543 and MG519671–MG519707.
