# Supplementary material for: Effects of environmental variables on abundance of ammonia-oxidizing communities in sediments of Luotian River, China
Source: PeerJ. 2020 Jan 6;8:e8256. doi: 10.7717/peerj.8256 (PMC6951284; doi:10.7717/peerj.8256)
Supplement: Supplemental Information 1 — Figure S1. Representation of phylogenetic relationships of ammonia-oxidizing bacteria (AOB) gene sequences through neighbor joining. Bootstrap supported values were 1000 replicates (values larger than 60% were indicated). The scale bar represents 0.2; evolutionary analyses were implemented on MEGA 7, Figure S2. Representation of Spearman correlation matrix was calculated between the gene copy numbers of 16S rRNA, AOA, and AOB and physicochemical parameters. The colors of the scale bar indicate the nature of the correlation with 1 denoting perfect positive correlation (green), and -1 denoting perfect negative correlation (red) were tested at p < 0.01 and p < 0.05. The used physicochemical data of the samples applied to Xlstat (www.xlstat.com), Table S1. The PCR primer sets and thermal profiles, Table S2. The gene copy numbers of 16S rRNA, AOA, and AOB in sampling sites. Values and standard deviations were estimated from triplicate an analysis within a single qPCR, Table S3. T-RF fragments and their corresponding clones of AOA. [file peerj-08-8256-s001.docx]

Supplementary Information

**Effects of Environmental Variables on Abundance of**

**Ammonia-oxidizing Communities in Sediments of**

**Luotian River, China**

Amjed Ginawi^1,2^, Lixiao Wang^1^, Wang Huading^1^, Yu Bingbing^1^, Yunjun Yan^1,*^

^1^ Key Lab of Molecular Biophysics of Ministry of Education, College of Life Science and Technology, Huazhong University of Science and Technology, 1037 Luoyu Road, Wuhan, 430074, China.

^2^ Faculty of Marine Science and Fisheries, Red Sea University, Port Sudan, Sudan.

[amjedginawi@rsu.edu.sd](mailto:amjedginawi@rsu.edu.sd) (A.G.); [zdwanglixiao@163.com](mailto:zdwanglixiao@163.com) (L.W.); [huadingwang@hust.edu.cn](mailto:huadingwang@hust.edu.cn%20)  (H.W.); [bingbingyu@hust.edu.cn (B.Y.).](mailto:bingbingyu@hust.edu.cn%20(B.Y.).%20)

***** Correspondence: yanyunjun@hust.edu.cn; Tel.: +86-27-87792213

**Table S1.** The PCR primer sets and thermal profiles

| **Target gene** | **Primer** | **Thermal program** | **Size (bp)** | **References** |
| --- | --- | --- | --- | --- |
| 16S rRNA | 27F 5’-AGAGTTTGATCCTGGCTCAG-3’  1492R 5’-(S)*ACGGCTACCTTGTTACGACT-3’ | 5 min at 95°C, one cycle  1min a 95°C, 45s at 57°C, 60s at 72°C, 30 cycles  8 min at 72°C, one cycle | 1500 | (Lane 1991) |
| Bacterial 16S rRNA gene- qPCR | 27F 5’-AGAGTTTGATCCTGGCTCAG-3’  16S-rtR 5’-GCTGCCTCCCGTAGGAGT-3’ | 10 min at 95°C, one cycle  60s at 95°C, 30 s at 60°C, 30 s at 72°C, 40 cycles, 8min at 72°C, one cycle | 310 | (Amann et al. 1990) |
| AOA | Arch *amoA* F: 5’-TAATGGTCTGGCTTAGACG-3’  Arch *amoA* R: 5’-GCGGCCATCCATCTGTATGT-3’ | 5 min at 95 °C, 35 cycles of 45 s at 94 °C, 60 s at 53 °C, and 60 s at 72 °C, followed by 15 min at 72 °C. | 635 | (Francis et al. 2005) |
| AOB | *amoA*-1F: 5’-GGGGTTTCTACTGGTGGT-3’  *amoA*-2R : 5’-CCCCTCTGGAAAGCCTTCTTC-3’ | 5 min at 95 °C, 35 cycles of 45 s at 94 °C, 45 s at 55 °C, and 45 s at 72 °C, and a final cycle consisting of 60 s at 60 °C and 10 min at 72 °C. | 491 | (Rotthauwe et al. 1997) |

*S= G or C, PCR: polymerase chain reaction, AOA: ammonia-oxidizing archaea, and AOB: ammonia-oxidizing bacteria.

**Table S2.** The gene copy numbers of 16S rRNA, AOA, and AOB in sampling sites. Values and standard deviations were estimated from triplicate an analysis within a triplicate qPCR.

|  | **May** | | | **August** | | | **October** | | |
| --- | --- | --- | --- | --- | --- | --- | --- | --- | --- |
| **Sampling Sites** | **16S rRNA (copies/μg of DNA extract)** | **AOA (copies/μg of DNA extract)** | **AOB (copies/μg of DNA extract)** | **16S rRNA (copies/μg of DNA extract)** | **AOA (copies/μg of DNA extract)** | **AOB (copies/μg of DNA extract)** | **16S rRNA (copies/μg of DNA extract)** | **AOA (copies/μg of DNA extract)** | **AOB (copies/μg of DNA extract)** |
| S1 | 1.56E+09 | 5.23E+08 | 4.64E+08 | 1.19E+09 | 4.79E+08 | 2.03E+08 | 1.98E+09 | 3.85E+08 | 4.63E+08 |
| S2 | 6.79E+08 | 5.03E+08 | 2.55E+08 | 6.79E+08 | 5.23E+08 | 3.24E+08 | 6.09E+08 | 5.21E+08 | 3.83E+08 |
| S3 | 6.17E+08 | 3.22E+08 | 3.36E+08 | 6.89E+08 | 2.23E+08 | 3.45E+08 | 6.04E+08 | 3.69E+08 | 3.25E+08 |
| S4 | 3.83E+08 | 3.19E+08 | 3.30E+07 | 3.87E+08 | 2.83E+08 | 3.60E+08 | 3.08E+08 | 3.92E+08 | 2.66E+08 |
| S5 | 7.42E+08 | 4.41E+08 | 5.45E+08 | 7.62E+08 | 4.25E+08 | 5.05E+08 | 7.40E+08 | 4.08E+08 | 4.97E+08 |
| S6 | 1.01E+09 | 5.27E+08 | 1.23E+08 | 1.25E+09 | 5.13E+08 | 1.08E+08 | 1.31E+09 | 5.22E+08 | 1.25E+08 |
| S7 | 6.51E+08 | 5.28E+08 | 4.79E+08 | 6.68E+08 | 5.02E+08 | 4.53E+08 | 6.07E+08 | 5.24E+08 | 4.49E+08 |
| S8 | 7.17E+08 | 4.00E+08 | 5.09E+08 | 7.79E+08 | 4.43E+08 | 5.05E+08 | 7.17E+08 | 4.68E+08 | 5.11E+08 |
| S9 | 5.85E+08 | 3.78E+08 | 4.56E+08 | 4.91E+08 | 3.82E+08 | 4.60E+08 | 5.85E+08 | 4.74E+08 | 4.58E+08 |

Table S3. Comparison of AOA and AOB diversities as well as coverage estimates in sampling sites base on 95% amino acid residue cutoff.

|  |  | **AOA** |  | **AOB** |
| --- | --- | --- | --- | --- |
| **Sampling sites** | **No. of clone sequenced** | **No. of OTUs^*^** | **No. of clone sequenced** | **No. of OTUs^*^** |
| S1 | 4 | 4 | 8 | - |
| S2 | 5 | 4 | 6 | - |
| S3 | 5 | 1 | 5 | - |
| S4 | 5 | 1 | 4 | - |
| S5 | 6 | 4 | 9 | - |
| S6 | 5 | 1 | 5 | - |
| S7 | 5 | 4 | 7 | 1 |
| S8 | 14 | 12 | 9 | 1 |
| S9 | 5 | 3 | 8 | 1 |
| Total | 54 | 34 | 61 | 3 |

^–^, undetected

^*^Unique OTUs of the *amoA* sequences were determined using the DOTUR program.

Table S4. T-RF fragments and their corresponding clones of AOA

| T-RF | Clone | Genus |
| --- | --- | --- |
| 108 | S6, S5, S9 | *Nitrososphaera* (3 clones) |
| 116 | S7, S8, S7  S8, S7, S8, S1 | *Nitrososphaera* (3 clones)  *Nitrosopumilus* (4 clones) |
| 237 | S1, S8, S2 | *Nitrososphaera* (3 clones) |
| 291 | S8, S8 | *Nitrososphaera* (2 clones) |
| 382 | S4, S4 | *Nitrosotalea* (2 clones) |
| 461 | S2, S8 | *Nitrososphaera* (2 clones) |
| 486 | S5, S6, S7, S9, S9, S8  S3, S5, S6, S5, S9 | *Nitrososphaera (6* clones)  *Nitrosopumilus* (5 clones) |
| Other | S2 | Uncultured ammonia oxidizing (1 clone) |

**References**

Amann RI, Binder BJ, Olson RJ, Chisholm SW, Devereux R, and Stahl DA. 1990. Combination of 16S rRNA-targeted oligonucleotide probes with flow cytometry for analyzing mixed microbial populations. *Applied and Environmental Microbiology* 56:1919-1925.

Francis CA, Roberts KJ, Beman JM, Santoro AE, and Oakley BB. 2005. Ubiquity and diversity of ammonia-oxidizing archaea in water columns and sediments of the ocean. *Proceedings of the National Academy of Sciences of the United States of America* 102:14683-14688. 10.1073/pnas.0506625102

Lane DJ. 1991. 16S/23S rRNA sequencing*.* In: Stackebrandt E , Goodfellow M (eds) Nucleic Acid Techniques in Bacterial Systematics. *Wiley,* New York, pp115–175.

Rotthauwe JH, Witzel KP, and Liesack W. 1997. The ammonia monooxygenase structural gene *amoA* as a functional marker: Molecular fine-scale analysis of natural ammonia-oxidizing populations. *Applied and Environmental Microbiology* 63:4704-4712.
