# Supplementary figures and images for: Effects of environmental variables on abundance of ammonia-oxidizing communities in sediments of Luotian River, China
Source: PeerJ. 2020 Jan 6;8:e8256. doi: 10.7717/peerj.8256 (PMC6951284; doi:10.7717/peerj.8256)

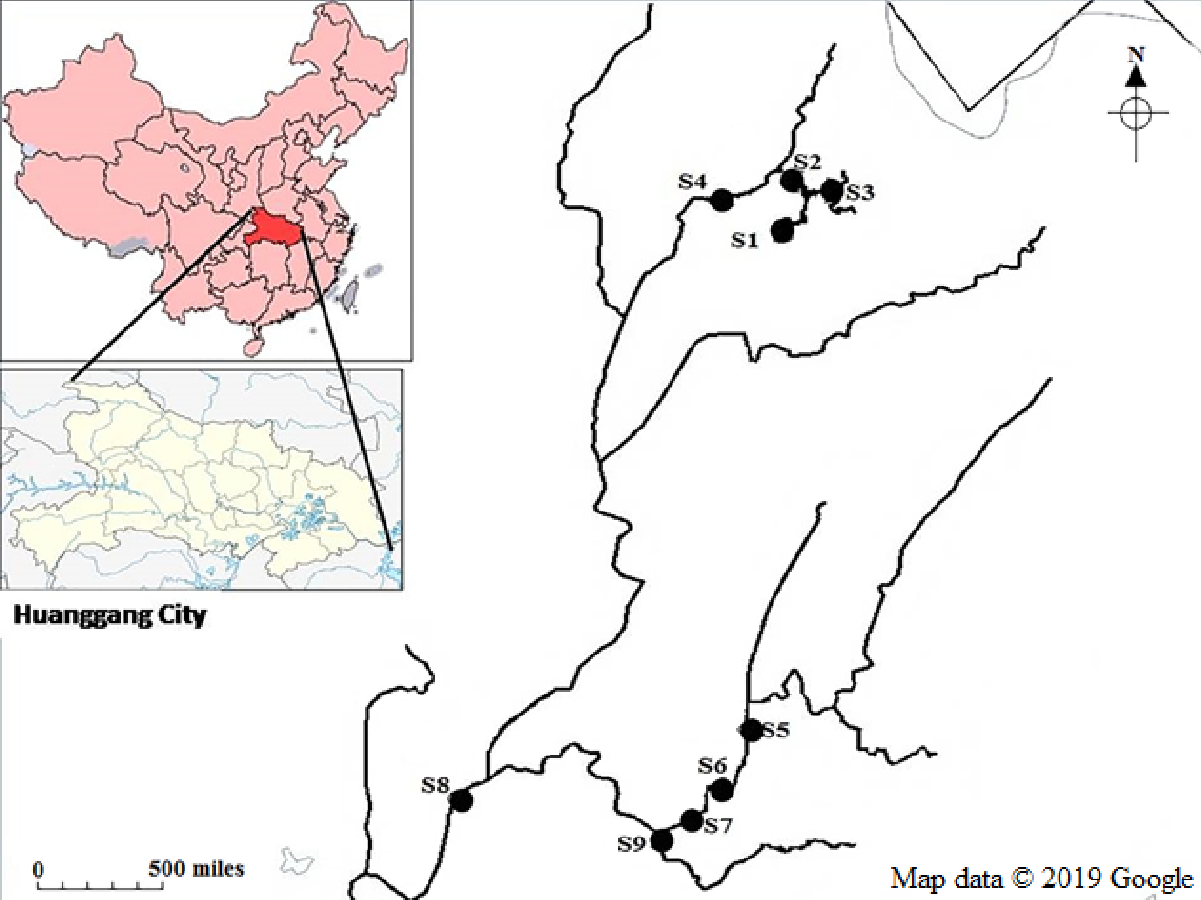

Supplement: Figure S1 — A schematic showing the locations of the sampling sites in the Luotian River; pristine upstream (S1–S4); human influences in Luotian city (S5–S7); downstream, located far from anthropogenic activities (S8); and discharge of waste treatment plant (S9), Map data ©2019 Google. [file peerj-08-8256-s002.png]

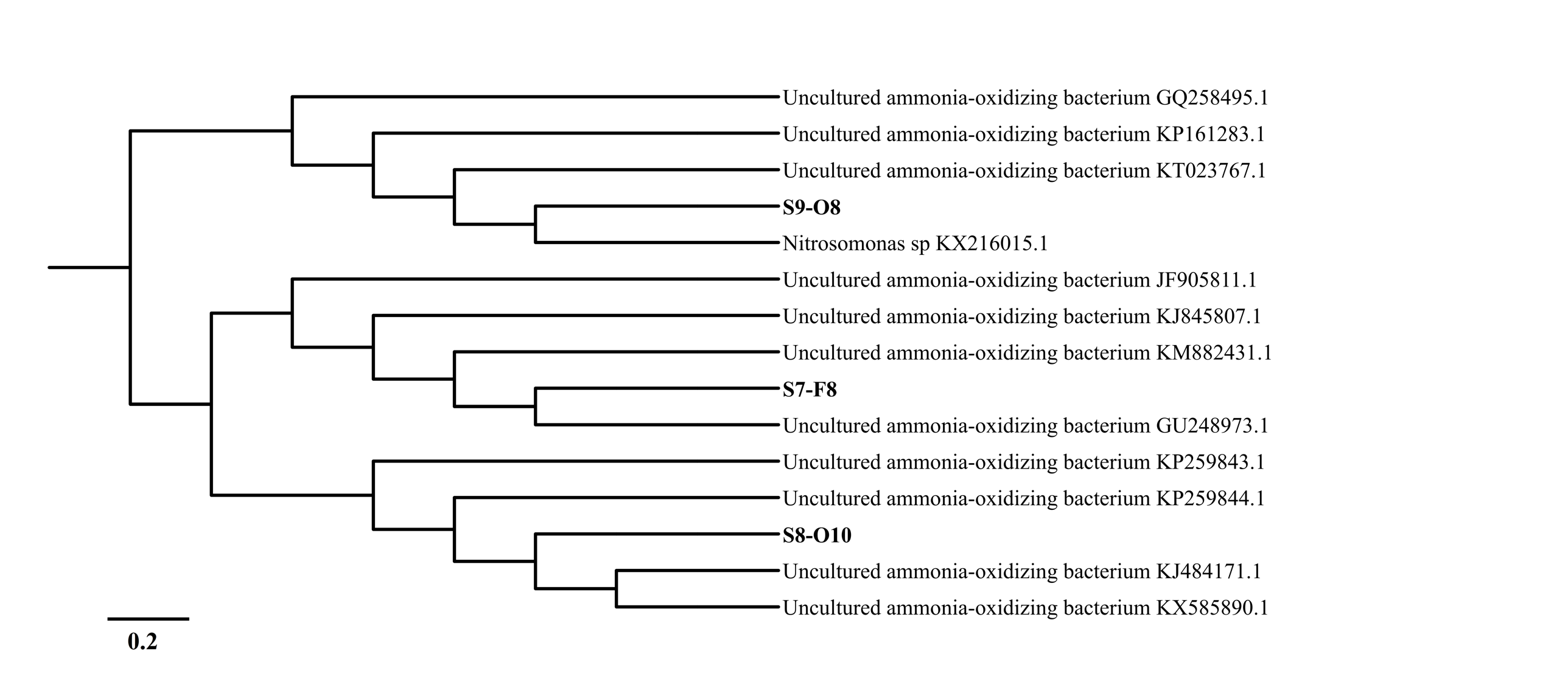

Supplement: Figure S2 — Representation of phylogenetic relationships of ammonia-oxidizing bacteria (AOB) gene sequences through neighbor joining. Bootstrap supported values were 1000 replicates (values larger than 60% were indicated). The scale bar represents 0.2; evolutionary analyses were implemented on MEGA 7. [file peerj-08-8256-s003.jpg]

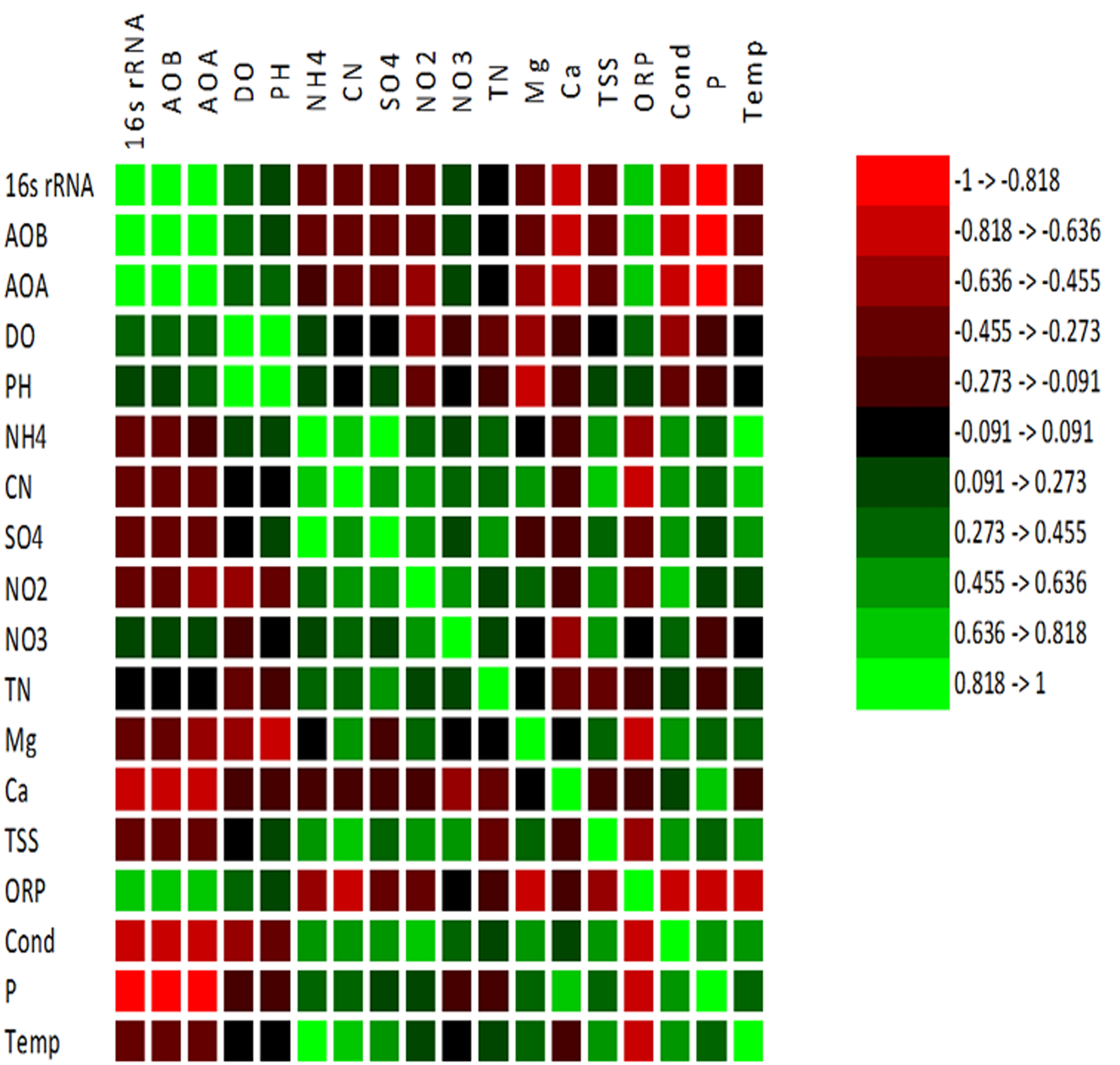

Supplement: Figure S3 — Representation of Spearman correlation matrix was calculated between the gene copy numbers of 16S rRNA, AOA, and AOB and physicochemical parameters. The colors of the scale bar indicate the nature of the correlation with 1 denoting perfect positive correlation (green), and -1 denoting perfect negative correlation (red) were tested at p¡0.01 and p¡0.05. The used physicochemical data of the samples applied to Xlstat (www.xlstat.com). [file peerj-08-8256-s004.jpg]
